# The effects of voluntary complex and regular wheel running exercises on the levels of 8‐oxoguanine DNA glycosylase, semaphorin 3B, H2O2, and apoptosis in the hippocampus of diabetic rats

**DOI:** 10.1002/brb3.1988

**Published:** 2021-01-20

**Authors:** Mohammad Fazelzadeh, Mohammad Esmaeil Afzalpour, Ziya Fallah Mohammadi, Hossein Falah Mohammadi

**Affiliations:** ^1^ Department of Exercise Physiology Faculty of Sport Sciences University of Birjand Birjand Iran; ^2^ Faculty of Sport Sciences Department of Exercise Physiology University of Mazandaran Babolsar Iran; ^3^ Faculty of Natural Sciences Department of Biology Ulm University Baden‐Württemberg Germany

**Keywords:** apoptosis, complex wheel running, OGG_1_, semaphorin 3B, voluntary exercise

## Abstract

**Purpose:**

One of the most frequent complications associated with diabetes mellitus is apoptosis within the brain which can lead to cognitive disorders. Exercise is considered the best non‐pharmacological approach to reduce the severity and extent of cell death through poorly‐understood mechanisms. The aim of this study was to investigate the effects of voluntary complex and regular wheel running on the levels of 8‐oxoguanine DNA glycosylase (OGG_1_), semaphorin 3B (sema3B), hydrogen peroxide (H_2_O_2_), and apoptosis in the hippocampus of diabetic rats.

**Methods:**

48 Wistar male rats were randomly divided into 6 groups: healthy control (C), diabetes control (D), regular wheel running + diabetes (RWD), complex wheel running + diabetes (CWD), healthy regular wheel running (RW), and healthy complex wheel running (CW). The diabetic rat model was produced by intraperitoneal injection of streptozotocin (STZ). The protocol encompassed a 4‐week voluntary running training regimen on regular and complex wheel running apparatus. The rats were sacrificed 48 hr after the last training session. To measure the protein concentrations within the hippocampus, ELISA has been utilized. One‐way ANOVA was used to compare the groups.

**Results:**

There were no significant differences in OGG1 protein levels between the groups. H_2_O_2_ level in the D group was significantly higher than the C group (*p* = .002), while this in RWD and CWD groups was considerably lower than the D group (*p* = .002 and *p* = .003, respectively). In the D group, the levels of apoptosis and Sema3B were significantly (*p* = .001 and *p* = .007, respectively) higher than C, RWD (*p* = .001, *p* = .0001, respectively), and CWD groups (*p* = .001, *p* = .006, respectively). Nevertheless, there were not any significant differences between RWD and CWD groups.

**Conclusion:**

The increased levels of Sema3B, H2O2, and apoptosis within the hippocampus associated with diabetes could be noticeably restored by both types of voluntary wheel running protocols.

## Graphical abstract

Performing both complex and voluntary wheel running protocols enhanced apoptotic indices in diabetic rat model. The increased levels of Sema3B, H2O2, and apoptosis within hippocampus associated with diabetes could be noticeably restored by both types of exercises. Therefore, exercise can be used as a safe therapeutic approach against negative impacts of diabetes on the brain.

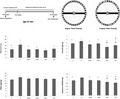

## 1 INTRODUCTION

Diabetes mellitus is a chronic and progressive metabolic disorder, and the complications associated with it account for 2.2% of all deaths in the world. Oxidative stress, an imbalance between production and detoxification of oxygen or nitrogen free radicals, contributes to the onset and progression of diabetes (Rains & Jain, 2011). It has been shown that free radicals increase the risk of diabetes‐related disorders such as endothelial dysfunction, cardiomyopathy, retinopathy, nephropathy, and neuropathy in diabetic patients (Zatalia & Sanusi, 2013). Diabetes causes neuronal apoptosis in the brain through several mechanisms, including oxidative stress, inhibition of caspases, disturbance in the expression of apoptotic regulator genes, as well as mitochondrial dysfunction. Any abnormalities within the apoptotic pathway may cause pathological conditions such as neurodegenerative diseases (Radi et al., 2014). Within the brain, the hippocampus is particularly susceptible to certain clinical conditions such as ischemia, stress, and diabetes leading to neurophysiological, structural, and molecular changes in this region. There have been studies showing a reduction in memory and learning capabilities following diabetes (Boulton et al., 2005).

Nuclear and mitochondrial DNA are the main targets of free radicals leading to cell death and apoptosis. 8‐hydroxy‐2′‐deoxyguanosine (8‐OHdG) is the most commonly considered biomarker of oxidative DNA damage, as it represents 5% of total oxidized bases in DNA and therefore can be easily detected (Helbock et al., 1999). The mutagenic base, 8‐OHdG, is a consequence of oxidative stress by hydroxyl radicals attacking 2'‐deoxyguanosine (dG) resulting in a hydroxyl moiety replacing the hydrogen atom (Van Remmen et al., 2003). The level of 8‐OHdG is remarkably increased in diabetic patients, and also in a rat model of diabetes (Nishikawa et al., 2003). Base excision repair (BE) is the major repair system for oxidative base damage, and this system is particularly crucial in neurons because of their high oxygen metabolism rate (Jeppesen et al., 2011). OGG1 is among the enzymes in this repair system that plays important roles. The OGG1 gene is located on short arm of chromosome 3 and encodes 8‐hydroxyguanine glycosylase, an enzyme which catalyzes the excision of 8‐OHdG (Zou et al., 2016). It has been reported that overexpression of cellular hydrogen peroxide (H2O2) decreases the expression and activity of the OGG1 enzyme, compromising its ability in protecting DNA (Kim et al., 2014). Induction of type I diabetes in rats has been shown to decrease OGG1 activity leading to higher risks of DNA damage in kidney cortical cells (Simone et al., 2008).

It has been demonstrated that axon‐guiding molecules, especially semaphorins, play critical roles in the formation of the vascular network as well as induction of apoptosis in neurons (Neufeld & Kessler, 2008; Shirvan et al., 1999). The biological form of this molecule in vertebrates is class 3 semaphorin (Neufeld & Kessler, 2008). One of the subunits of class 3 semaphorin, Sema3B, has been particularly known for its role in the induction of apoptosis within nerve cells in certain pathological conditions, and thus its up‐regulation induces apoptosis and suppression of cancerous tumors (Shirvan et al., 1999). It has been reported that Sema3A is highly expressed in patients with diabetic nephropathy, which can lead to apoptosis within kidney podocytes, and thereby accelerating disease progression (Aggarwal et al., 2015). As a result, we hypothesized that there is an increased level of Sema3B and a decreased level of OGG1 in diabetic patients, which would increase the extent of apoptosis in the brain. Therefore, restoring the levels of these two molecules through exercise as a safe and non‐pharmacological approach might potentially decrease the adverse effects of diabetes on the brain.

Although there are several contradictory studies concerning the effects of exercise on antioxidant enzyme activities, exercise can indeed improve brain functions by balancing oxidation–regeneration processes through increasing resistance against oxidative stress and accelerating the recovery from its detrimental effects (Devi & Kiran, 2004; Radak et al., 2007). It has been shown that even highly intensive exercises are unlikely to cause significant oxidative damage to DNA (Ogonovszky, Berkes, et al., 2005). It has been reported that regular exercise reduces the accumulation of 8‐OHdG in the hippocampus (Mahjoub et al., 2016). This is while, the production of moderate amounts of 8‐OHdG is important for neurogenesis (Walton et al., 2012). Exercise has been shown to be able to modulate the activity of DNA repair enzymes, especially OGG1 and uracil DNA glycosylase (UDG), thereby reducing 8‐OHdG accumulation and mutation in skeletal muscle cells (Sallam & Laher, 2016). Similarly, Bo et al. (2014) have shown that performing prolonged treadmill running exercise restores the impaired activity of OGG1 and the mitochondrial antioxidant enzymes within the hippocampus in rats model of Alzheimer`s disease (Bo et al., 2014). Although there are reports about the positive effects of treadmill running and swimming exercises on the base excision repair system, and OGG1 and UDG enzymes, there are not enough studies investigating the effects of voluntary wheel running on the levels of OGG1 and UDG enzymes (Sallam & Laher, 2016).

Wheel running is a type of endurance exercise resembling the normal physical activity in rats and humans, with minimal coercion and injury, while ensuring adequate training intensity (Sun et al., 2016). Unlike forced treadmill running, performing voluntary wheel running exercise does not overstimulate stress hormones, and thus maintains healthy cognitive capabilities. However, Toval et al. (2020) suggested that the implementation of an adaptive phase prior to performing forced exercise protocols might prevent non‐specific stress responses (Toval et al., 2020). Voluntary exercise has been shown to exert beneficial effects on the functions of the hippocampus dentate gyrus. It has been demonstrated that optional regular wheel running results in a three‐ to four‐fold increases in the production and survival of newly‐born neurons in the hippocampal dentate gyrus (van Praag, 2009). According to Sun et al. (2016), while optional training noticeably increases the mRNA expression levels of Bcl‐XL anti‐apoptotic protein in rat skeletal muscle mitochondria, it does not change the expression levels of Bax apoptotic protein, and Bcl‐2/Bax expression ratio (Sun et al., 2016). Furthermore, it has been shown that four weeks of exercise has positive effects on the brain and cognitive functions (Nouchi et al., 2014). Exercises that engage cognition and creativity may have more effective consequences; therefore, a complex wheel running apparatus consisting of stairs with irregular gaps have been designed which, unlike regular wheel running, would make running more challenging and trickier for rats. Consequently, in addition to performing physical activity, the cognitive functions will be engaged as the rats must adjust their running rhythm continuously (McKenzie et al., 2014). The rhythmic locomotion during the regular wheel running is largely controlled by the central pattern generator within the spinal cord, a process that is triggered by a feedback loop between deep sensory and nerve afferents (Rossignol et al., 2006). In this context, the supraspinal motoric output would be less important in the regular wheel running. In contrast, continuous step‐length adaptation and bilateral coordination during complex wheel running necessitate substantial involvement of supraspinal circuits (Liebetanz et al., 2007). McKenzie et al. (2014) have shown that there is an increased production of myelin‐forming oligodendrocytes in mice that have acquired novel skills during running on complex rotating wheel apparatus (McKenzie et al., 2014). Liebetanz and Merkler (2006) reported that the remaining impairments of Vmax in remyelinated neurons of mice on the complex wheel apparatus possibly reflect latent supraspinal deficiencies of motor coordination that are not recognizable on the regular wheels (Liebetanz & Merkler, 2006). Therefore, the aim of this study was to investigate and compare the effects of voluntary complex and regular wheel running on the levels of OGG1, sema3B, H2O2, and apoptosis in the hippocampus of diabetic rats. Hence, the authors have explored the causal mechanisms of cell death survival after voluntary exercise in diabetic rats.

## 2 METHODS

### 2.1 Animals and experimental design

48 adult male Wistar rats (8–10 weeks of age, and 235 ± 10 g) were randomly divided into 6 groups (*n* = 8): 1. Healthy control (C), 2. Diabetic control (CD), 3. Regular wheel running + diabetes (RWD), 4. Complex wheel running + diabetes (CWD), 5. Healthy regular wheel running (RW), and 6. Healthy complex wheel running (CW). The study was performed according to the principles of the Declaration of Helsinki, and all the procedures involving animal experiments were approved by the ethical committee of the University of Mazandaran, Iran.

The diabetic rat model was produced by a single intraperitoneal injection of streptozotocin (STZ) (50 mg/kg body weight) diluted in citrate buffer in the second week of the study. 72 hr following STZ injection, the blood samples were collected from the tail vein to determine the levels of blood glucose using a glucometer (ACCU‐CHEK Active, Germany). Glucose concentration higher than 250 mg/dl was considered as diabetic (Jafari Anarkooli et al., 2014).

**FIGURE 1:**
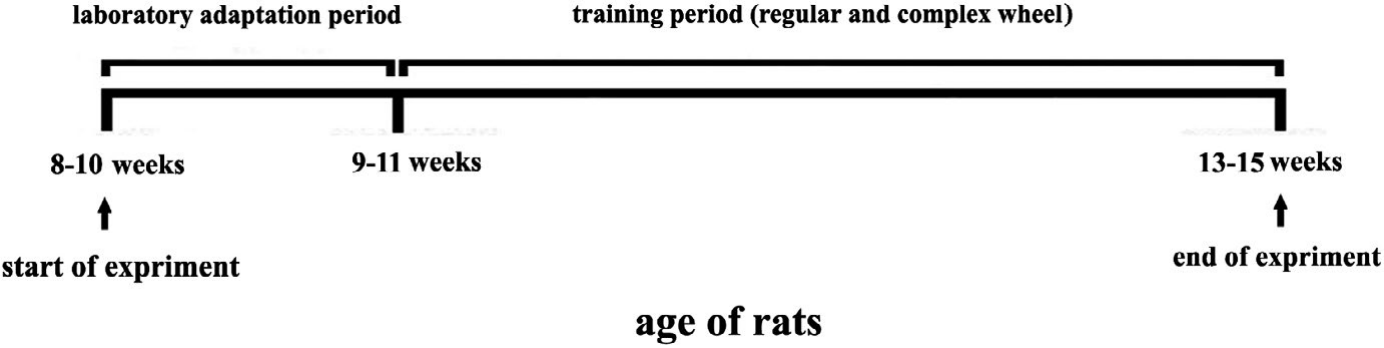
Experimental design of this study. 8‐ to 10‐week‐old animals were adapted to laboratory condition for one week. Then the diabetic model was produced using a single intraperitoneal injection of 50 mg/kg STZ. The training protocols were performed over a period of four weeks

**FIGURE 2:**
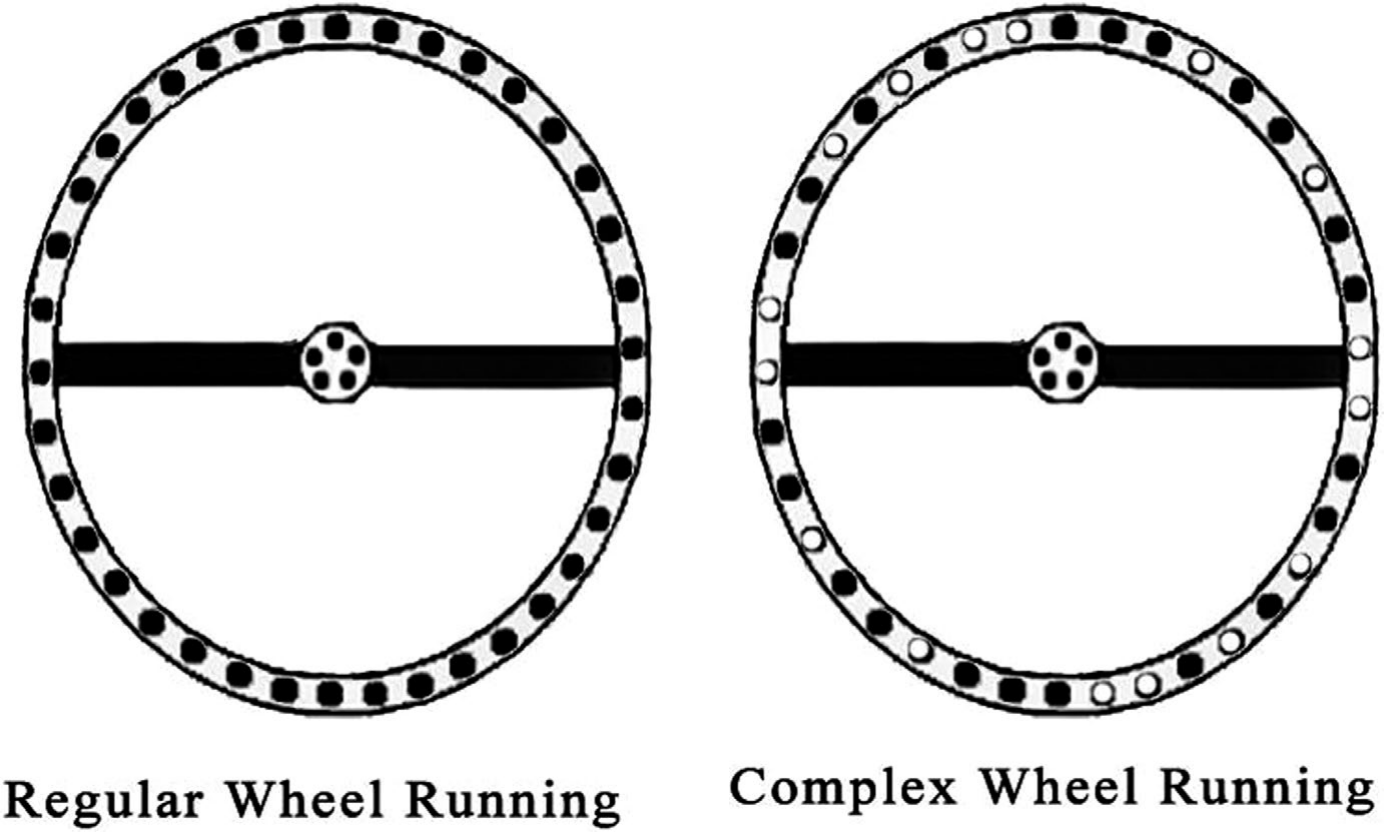
Two types of running wheel systems used in this study. The regular wheel running apparatus is made of 38 steps with regular intervals (filled circles) in between. The complex wheel running apparatus comprises 22 steps with irregular gaps (empty circles) between each

The rats were kept in the animal laboratory of the faculty of sports sciences at the University of Mazandaran at a standard condition, the temperature of 22 ± 2ºC with a relative humidity of 50%, and the 12:12 hr light/dark cycle. Moreover, the animals had ad lib access to food and water. Rats in the voluntary exercise groups were individually housed in cages with stainless‐steel running wheel apparatus (manufactured by the faculty of sports sciences, university of Mazandaran), and they had free access to the wheels all day long for four weeks (Figure 1). The regular wheel running apparatus is made of 38 steps with regular intervals in between. However, the complex wheel running apparatus is comprised of 22 steps with irregular gaps between each. The pattern of stairs in a complex wheel apparatus has been displayed in Figure 2 (McKenzie et al., 2014). Running distance was monitored and recorded daily. Also, the sedentary rats were housed in standard holding cages without running wheel apparatus for the same period of time.

### 2.2 Tissue isolation and preparation

At the end of the 4th week, and 48 hr after the last training session, the rats were anesthetized with an intraperitoneal injection of ketamine (80 mg/kg) and xylazine (8 mg/kg). Subsequently, the rats were decapitated, the brain was removed, and the hippocampus was isolated. The tissues were then immediately snap‐frozen in liquid nitrogen at −80°C. Hippocampal tissue was physically homogenized in liquid nitrogen. Finally, the samples were centrifuged at 6,000 rpm for 15 min. Then, the supernatant was used for further measurements.

### 2.3 Biochemical assays

Apoptosis levels of the hippocampus were measured with cell death detection ELISA kit which enables assessment of histone‐associated DNA fragments. All the procedures have been performed according to the manufacturer's instructions (Roche Molecular Biochemicals, Germany). Also, we used the commercially available ELISA kit (ZellBio GmBH, Germany) to measure the concentrations of OGG1, and Sema3B proteins within the hippocampus according to the manufacturer's protocol. For measuring H2O2 activity in the hippocampus, a Hydrogen Peroxide Colorimetric Assay Kit has been utilized (ZellBio GmBH, Germany). The specificity of the kits was less than 0.01, 0.03 ng/ml, and 5 µM for OGG1, Sema3B, and H2O2, respectively. Data were extrapolated using an ELISA reader at 570 nm (Biotech, USA), and presented as mg tissue/weight.

### 2.4 Statistical analysis

Shapiro–Wilk's and Levene's tests were used to test the normality and equality of variances, respectively. One‐way analysis of variance with Tukey's post hoc test was used to compare the intergroup differences. An independent *t* test was also used to compare the running distance between the RWD and CWD groups. A P‐value of less than 0.05 was considered as statistically significant. All the statistical analysis and plotting graphs were performed using SPSS software (version 19.0) and presented as means ± standard deviation (*SD*).

## 3 RESULTS

### 3.1 Weight and mileage

The mean and *SD* of body weight and running distance are displayed in Table 1. The data revealed that at the end of the experiment, the weight of rats in D, RWD, and CWD groups was significantly lower than that of the C, RW and, CW groups **(*p* = .0001, *p* = .0001, *p* = .0001, respectively).** However, 4 weeks of performing exercise protocols did not lead to any significant differences in the weight of RWD and CWD groups compared to the D group (*p* > .05). Overall, the diabetic rats have run shorter distances compared to the healthy rats. Furthermore, there was no significant difference in weight and mileage between the RWD and CWD groups (*p* > .05).

**TABLE 1:** Mean and standard deviation of weight and mileage among different groups

Groups						Variables
CW	RW	CWD	RWD	D	C	
320.7 ± 31.1	340.7 ± 39.1	230.3 ± 32*	221.8 ± 34.5*	202.2 ± 21.4*	315.7 ± 28.3	Body weight (gr)
37,687 ± 16,686	37,396 ± 1,270	13,125 ± 4,802**	12,331 ± 5,803**	—	—	Mileage (m)

* Significant difference between D, RWD, and CWD groups with C, RW, and CW groups (*p* < .05).

** Significant difference between RWD, and CWD groups with RW, and CW groups (*p* < .05).

### 3.2 STZ‐induced diabetes in rats

The diabetic model was generated through a single intraperitoneal injection of 50 mg/kg STZ in the second week. To confirm diabetes in rats, 72 hr following STZ injection, the blood glucose levels were measured using a glucometer. Blood glucose levels increased significantly 72 hr following STZ injection. The blood glucose level was 92.37** ± **7.657 mg/dl in the control group and 373.8 ± 41.44 mg/dl in the diabetes group, which clearly demonstrates the induction of diabetes in rats. Glucose concentration higher than 250 mg/dl was considered as diabetic.

### 3.3 Performing running exercise decreases H2O2 levels in the hippocampus of diabetic rats to healthy levels

**FIGURE 3:**
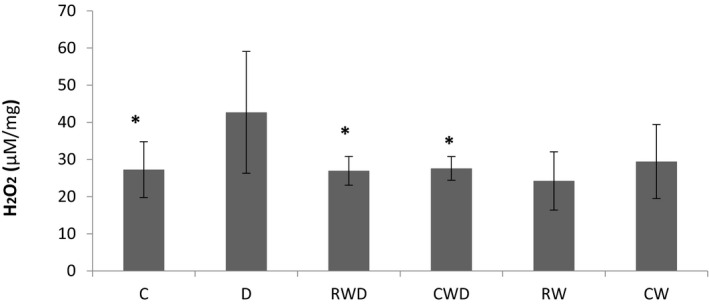
Comparing H2O2 levels between the groups using one‐way ANOVA. *Significant difference between C, RWD, and CWD groups with D group (*p* = .002, *p* = .002, and *p* = .003, respectively)

One‐way ANOVA showed that within the hippocampus, the level of H2O2 in the D group was significantly higher than that of the C group (*p* = .002). Besides, the concentration of H2O2 in RWD (*p* = .002) and CWD groups (*p* = .003) was significantly lower in comparison to the D group. The levels of H2O2 were comparable under both training conditions, suggesting similar potentials of both training protocols in reducing H2O2 (Figure 3). Overall, wheel running did not cause significant changes in H2O2 levels in healthy exercise groups compared to the C group.

### 3.4 Both running protocols induce beneficial effects on Sema3B and OGG1 protein concentrations in diabetic rats

**FIGURE 4:**
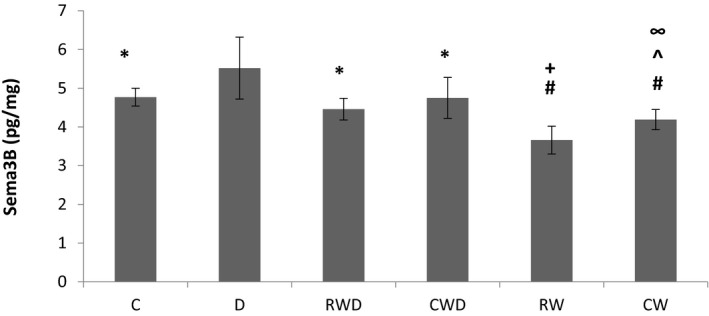
Comparison of Sema3B protein levels between the groups using one‐way ANOVA. * Significant difference between C, RWD, and CWD groups with D group (*p* = .007, *p* = .0001, and *p* = .006, respectively). # Significant difference between RW and CW groups with C group (*p* = .0001, and *p* = .03, respectively). + Significant difference between RW group with RWD group (*p* = .001). ^ Significant difference between CW group with CWD group (*p* = .03). ∞ Significant difference between CW group with RW group (*p* = .01)

**FIGURE 5:**
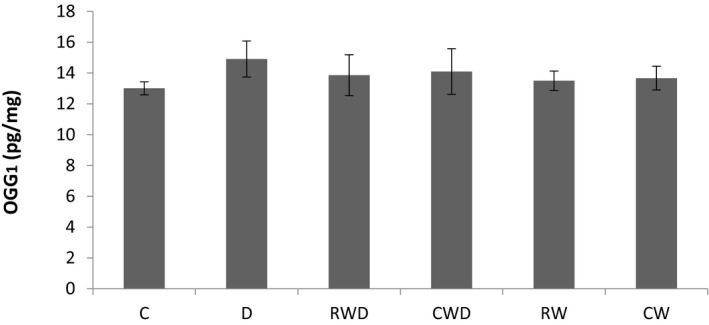
Comparing OGG1 protein levels between the groups

Our results have revealed that the Sema3B level in the hippocampus is quite similar to the pattern observed in H2O2. While the levels of Sema3B in D group were significantly higher than the C group (*p* = .007), it was considerably lower in RW and CW groups compared to C group (*p* = .0001, and *p* = .03, respectively). Furthermore, the Sema3B concentrations in RWD (*p* = .0001) and CWD groups (*p* = .006) were significantly decreased when compared to the D group, and were significantly higher than CW (*p* = .03) and RW (*p* = .001) groups. Although the level of Sema3B in the CWD group was slightly higher than the RWD group, it was not significant (Figure 4). As can be seen in Figure 5, there were not any significant differences in OGG1 protein concentrations between the groups (*p* > .05), although RWD and CWD groups showed marginally lower levels in comparison to that of the D group.

### 3.5 Exercise reduces the extent of cell death within the hippocampus of diabetic rats

**FIGURE 6:**
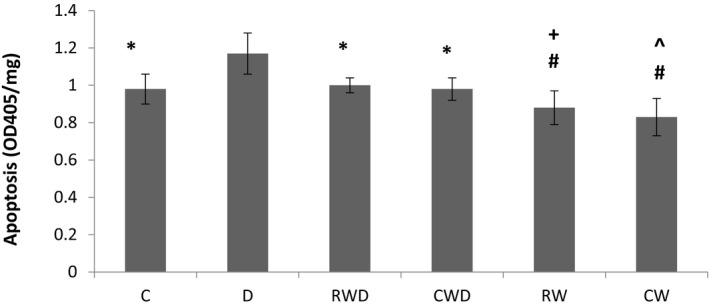
Comparing apoptosis rate between the groups. *significant difference between C, RWD, and CWD groups with D group (*p* = .001, *p* = .001, and *p* = .001, respectively). # Significant difference between RW and CW groups with C group (*p* = .03, and *p* = .005, respectively). + Significant difference between RW group with RWD group (*p* = .008). ^Significant difference between CW group with CWD group (*p* = .001)

A comparison of apoptosis extent within the hippocampus between each group showed that in the D group, there is a considerably higher rate of cell death compared to the C group (*p* = .001) (Figure 6). However, the apoptosis rate in RW and CW groups was lower than the C group (*p* = .03, and *p* = .005, respectively). One‐way ANOVA revealed that the apoptosis rate in RWD and CWD groups was almost identical to that of the C group manifested by a significant reduction of cell death in these groups compared to the D group (*p* = .001, and *p* = .001, respectively). Moreover, the level of cell death in RW and CW groups was significantly decreased compared to RWD (*p* = .008) and CWD (*p* = .001) groups respectively. Nevertheless, there was not any significant difference in apoptosis level between RWD and CWD groups. Altogether, the results suggest a similar capacity of performing both types of running exercises in exerting beneficial effects on the survival rates of cells within the hippocampus.

## 4 DISCUSSION

In this study, we have demonstrated that apoptosis is increased within the hippocampus of diabetic rats following STZ injection, which has been characterized by significant increases in H2O2 and Sema3B levels in this region. We have shown that both regular and complex voluntary wheel running protocols are able to restore the levels of these molecules almost back to normal.

Accordingly, Pamidi and Nayak (2014) reported that 30 days after injecting STZ, the number of survived hippocampal neurons were significantly decreased compared to that of the control group (Pamidi & Nayak, 2014). They have shown that the increased levels of apoptosis observed in experimental diabetes mellitus models are directly associated with the levels of free radicals and oxidative stress. Similarly, several other studies have reported increased levels of H2O2 in the brain of diabetic rats. It has been demonstrated that hyperglycemia associated with diabetes leads to increased H2O2 production and oxidative stress through activating NADPH oxidase (Zabielski et al., 2016). Prominently, oxidative stress and inflammation have been shown to be linked to high production of adhesion molecules and pro‐inflammatory cytokines such as IL‐6 and TNF‐α, and thereby increasing the risk of diabetes. Diabetes is a chronic pro‐inflammatory condition that leads to reductions in the intracellular antioxidant reserves decreasing the ability of cells to resist oxidative stress. In fact, high concentrations of reactive oxygen species (ROS) can damage the DNA which can lead to cell death via direct oxidation, or by compromising repair mechanisms (Federico et al., 2007).

In this study, OGG1 protein levels did not change significantly in diabetic rats after 4 weeks of performing running training protocols. Simone et al. (2008) showed that 4 weeks following intravenous administration of 55 mg/kg STZ, there was a decreased level of OGG1 in rat kidney (Simone et al., 2008). It has been demonstrated that STZ injection at high doses (150 mg/kg) increases the levels of 8‐OHdG and OGG1 in skeletal muscle of diabetic rats (Sriram et al., 2015). Interestingly, it has been reported that 10 weeks after injecting 40 mg/kg STZ, the activity of the OGG1 enzyme in mice heart reduced noticeably, whereas the levels of OGG1 were increased (Cividini et al., 2016). These inconsistencies may be due to the varied evaluation of the activity, expression, and protein levels OGG1. Also we cannot exclude the type of subjects and tissues that were examined, and the dose and mode of STZ injection, the length of the study period, and the time after the induction that was analyzed could be among other factors affecting the findings. Unlike previous studies, here, we only measured the concentration, but not the activity and expression levels of OGG1. Furthermore, the levels of 8‐OHdG were not evaluated in this study, which could have also shown variability as in the above‐mentioned studies. Among the DNA bases, the guanine is particularly susceptible to oxidation, and up until now more than 20 oxidized products of this base have been identified, of which 8‐OHdG is the most abundant (Nishimura, 2002). The concentration of 8‐OHdG above the physiological levels results in increased levels of OGG1, which could consequently eliminate 8‐OHdG from DNA (Nishimura, 2002). The present study did not measure 8‐OHdG levels, but, likely, it did not increase significantly 4 weeks after induction of diabetes, and thus no significant changes in OGG1 levels can be expected. Another possible reason that the OGG1 levels did not raise following STZ injection lies behind the possible increases in the activities of antioxidant enzymes in the hippocampus which protects DNA against the STZ‐induced free radicals invasion, and there is no need for high levels OGG1.

As already mentioned, semaphorin 3B is an important factor playing roles in the initiation of apoptosis. So far, there have not been many studies investigating the effects of Sema3B on diabetes. However, the association of other class 3 semaphorins, including Sema3A and Sema3E with diabetes has already been reported (Aggarwal et al., 2015; Shimizu et al., 2013). A few studies have demonstrated that inflammation and oxidative stress are regulatory factors of semaphorins (Joyal et al., 2011). Furthermore, it has been shown that there is an interaction between class 3 semaphorins (Sema3A) and oxidative stress, suggesting that oxidative stress could possibly be responsible for modulating the detrimental impacts of Sema3A on the kidney of diabetic mice (Mohamed et al., 2014). Morinaka et al. reported that Sema3A signaling locally enhances H2O2 concentration in dorsal root cones of nerve ganglia by activating MICAL1 and MICAL3 (Morinaka et al., 2011). Another protein that may play role in the induction of class 3 semaphorins, particularly Sema3B, is tumor suppressor protein, P53, although the precise underlying mechanism behind its regulation by the semaphorins is still elusive. It has been shown that overexpression of Sema3B through activation of caspase‐3 in the presence or even absence of p53 can activate the apoptotic pathway in cancer cells (Castro‐Rivera et al., 2004). Furthermore, a significant role for secretory semaphorins in regulating neuronal cell death has already been implicated, although their underlying mechanisms have not been elucidated yet (Gagliardini & Fankhauser, 1999). Moretti et al. (2008) have demonstrated that Sema3A signaling controls Fas‐induced apoptosis, CD95, through the transport of Fas into lipid rafts (Moretti et al., 2008). In this study, we have shown that the increased levels of H2O2 and Sema3B are associated with elevated levels of diabetes‐induced apoptosis in the brain.

Our results revealed that performing a 4‐week voluntary wheel running exercise on complex and regular apparatus results in a slight decrease in OGG1 concentration while inducing significant decreases in the levels of H2O2, Sema3B, and hippocampal apoptosis in diabetic rats. The intensity of exercise is one of the major factors affecting OGG1 and 8‐OHdG levels. It has been demonstrated that low‐ to moderate‐intensity swimming and running exercises do not have considerable effects on the levels of these molecules in the hippocampus of rats (Koltai et al., 2011). Moreover, reports are indicating the increases in OGG1 concentration in the brain and liver following performing extremely intensive endurance training (Ogonovszky, Sasvári, et al., 2005). As already mentioned, the intensity of training protocols utilized in this study is lower than that of forced exercises such as treadmill running or swimming. This explains the lack of noticeable changes in OGG1 concentration after performing exercises in this study. In addition, since OGG1 levels did not change significantly following the induction of diabetes, performing voluntary running exercise may not have a significant effect on its levels as well.

As previously mentioned, performing an exercise, on the one hand, enhances cognitive function, and on the other hand, accelerates nerve recovery after brain injury (Radak et al., 2006). Van Praag (2009) reported that performing voluntary wheel running exercise increases the production and survival of newly born neurons in the hippocampus dentate gyrus by 3–4 times (van Praag, 2009). Regular exercise probably modulates apoptosis through modifying the activity and gene expression of several principal apoptotic components. It has been shown that performing 10 days of voluntary exercise up‐regulates the anti‐apoptotic protein Bcl‐2 while down‐regulating the pro‐apoptotic protein Bax in the hippocampus of morphine‐dependent rats, which, simultaneously, enhances their cognitive performance (Mokhtari‐Zaer et al., 2014). However, more studies are required to unravel the exact mechanisms behind the effect of voluntary running training on apoptosis in the brain.

So far, there are not enough studies investigating the effects of exercise on semaphorins. In this study, we have shown that performing 4 weeks of voluntary exercise reinstated the increased concentration of Sema3B in the hippocampus of diabetic rats, the mechanism of which is yet to be elucidated. However, due to the interaction between oxidative status and semaphorins, it is possible that voluntary exercise decreases Sema3B levels by reducing oxidative stress and free radicals such as H2O2.

Interestingly, in this study, we discovered that there were no significant differences between the effects of the two types of exercise protocols used in this study in diabetic rats. This could be explained by the fact that at the beginning of the training session on a complex wheel apparatus, the perceptual elements of the rat's nervous system were involved in order to detect the irregularities between the stairs. Nevertheless, after several sessions, the rats discovered and got used to the running pattern, and thus the discrepancy between the two training groups in terms of cognitive performance became rather unrecognizable.

Overall, further studies should focus on investigating the difference between the two types of exercise in the short term and before the running pattern for animals becomes repetitive. Moreover, in order to challenge the cognition and creativity of rats, new protocols could be established in which the pattern of stair intervals after a short period of time would be altered. Also, in order to determine the level of exercise necessary to obtain the observed responses it would be crucial to explore forced exercises (treadmill or motorized wheels), because forced exercise ensures the control of exercise load that will determine the level of physical activity necessary for the observed and induced changes (Toval et al., 2017). Furthermore, it would be interesting to perform the protocols on animals of different ages, to investigate any changes that could be associated with different periods of life. In addition, other methods, such as morphological changes, terminal deoxynucleotidyl transferase dUTP nick end labeling (TUNEL), and caspase activity, should be employed to further identify the apoptotic cells.

In this study, we have demonstrated that apoptosis is associated with increased levels of Sema3B and H2O2 in the hippocampus and that both voluntary complex and regular wheel running exercise protocols were able to restore the levels of these molecules, and help the brain to at least in part recover from the adverse impacts of diabetes, and probably reduce the extent and severity of cell death in the hippocampus in affected individuals. However, more studies are required to substantiate the anti‐apoptotic effects of exercise by the means of various molecular and behavioral tests, and our study was only a preliminary contribution toward showing that exercise could potentially be considered as a side‐effect‐free non‐pharmacological therapeutic approach against diabetic‐induced apoptosis in the brain.

## CONFLICT OF INTEREST

The authors declare that there are no conflicts of interest. The results of the study are presented clearly, honestly, and without fabrication, falsification, or inappropriate data manipulation.

## AUTHOR CONTRIBUTION

Mohammad Fazelzadeh, Mohammad Esmaeil Afzalpour, and Ziya Fallah Mohammadi designed and conceptualized the study. Mohammad Fazelzadeh mainly contributed to the acquisition of data. All the authors contributed to the analysis and interpretation of data equally. Mohammad Fazelzadeh and Hossein Falah Mohammadi drafted the manuscript, and all the authors reviewed the drafted manuscript.

### Peer Review

The peer review history for this article is available at https://publons.com/publon/10.1002/brb3.1988.

## Data Availability

The data that support the findings of this study are available from the corresponding author upon reasonable request.
